# Lightweight Football Motion Recognition and Intensity Analysis Using Low-Cost Wearable Sensors

**DOI:** 10.1155/2023/2354728

**Published:** 2023-07-12

**Authors:** Qian Xie, Ning Jin, Shanshan Lu

**Affiliations:** ^1^College of Asia Heart Hospital Nursin, Wuhan Institue of Design and Sciences, Wuhan 430205, China; ^2^College of Sports, South-Central Minzu University, Wuhan 430074, China; ^3^College of Computer Science, South-Central Minzu University, Wuhan 430074, China

## Abstract

In recent years, machine learning has been utilized in health informatics and sports science. There is a great demand and development potential for combining the Internet of Things (IoT) and artificial intelligence (AI) to be applied to football sports. The conventional teaching and training methods of football sports have limited collection and mining of real raw data using wearable devices, and lack human motion capture and gesture recognition based on sports science theories. In this study, a low-cost AI + IoT system framework is designed to recognize football motion and analyze motion intensity. To reduce the communication delay and the computational resource consumption caused by data operations, a multitask learning model is designed to achieve motion recognition and intensity estimation. The model can perform classification and regression tasks in parallel and output the results simultaneously. A feature extraction scheme is designed in the initial data processing, and feature data augmentation is performed to solve the small sample data problem. To evaluate the performance of the designed football motion recognition algorithm, this paper proposes a data extraction experimental scheme to complete the data collection of different motions. Model validation is performed using three publicly available datasets, and the features learning strategies are analyzed. Finally, experiments are conducted on the collected football motion datasets and the experimental results show that the designed multitask model can perform two tasks simultaneously and can achieve high computational efficiency. The multitasking single-layer long short-term memory (LSTM) network with 32 neural units can achieve the accuracy of 0.8372, F1 score of 0.8172, mean average precision (mAP) of 0.7627, and mean absolute error (MAE) of 0.6117, while the multitasking single-layer LSTM network with 64 neural units can achieve the accuracy of 0.8407, F1 score of 0.8132, mAP of 0.7728, and MAE of 0.5966.

## 1. Introduction

With the rapid development of next-generation information technologies such as the Internet of Things (IoT) and artificial intelligence (AI), motion recognition based on wearable sensors has become a hot research topic, due to the high computing power, small size, and low cost of sensors [1]. The existing research is mainly applied to medical monitoring, human fall detection or gesture recognition, and other scenarios, focusing on the recognition of running, jumping, falling, and other common movements. However, the research of movement recognition in various sports is still in its infancy. The application of human activity recognition technology in various sports disciplines can help coaches objectively and efficiently judge the level of students. Taking football as an example, it is the premise of excellent performance for football players to skillfully and accurately grasp the basic actions such as passing and shooting, and these basic actions are also the focus of daily teaching and training. The combination of IoT and AI technologies in football teaching, training, and even matches have great application demand and development potential. Football motion recognition and analysis is mainly aimed at capturing and evaluating athletes' calves and ankles. Wearable devices with built-in inertial sensors are tied to the ankles, and learning algorithms are used to identify movement and estimate movement intensity.

Activity recognition and analysis of football is mainly used to capture and evaluate the calves and ankles of athletes. Before exercise, the wearable device with a built-in inertial sensor is tied to the ankle, and the machine learning algorithm is used to identify movements and estimate movement intensity. In general, the sensitivity of inertial sensor to the data capture of lower body motion movements is low, especially the football motion movements are complex and the individual athletes are different. The existing algorithm model of human activity recognition is difficult to be directly used in football motion recognition. In the traditional football teaching and training methods, the collection and mining of real raw data is limited, lacking the human motion capture and posture recognition based on sports science theory. The current model is not deep enough in the analysis of the action data flow of complex and high noise.

## 2. Related Work

In recent years, wearable sensors or devices have been widely applied to motion capture and recognition domains [2]. These applications include but are not limited to intelligent visual monitoring, such as abnormal behavior detection in public places and intelligent medical facilities, daily activity monitoring, and fall detection in nursing homes. As for the image action recognition technology in human action recognition, the main research task is to detect abnormal events through the detection of actions, not necessarily to learn and classify different types of motions. Although image analysis techniques can be used in the sports research field to make more accurate identification of athletes' behavior. However, this technology has some disadvantages in the development of the motion scene: the equipment cost is expensive, the system is vulnerable to the influence of the external environment, and relevant visual algorithms and image processing depend on a large number of hardware conditions [3]. With the popularity of IoT applications, sensor technology is developing rapidly, among which inertial microelectromechanical systems (MEMS) sensors are the most widely used. Its advantages mainly lie in its sensitive induction, appropriate induction range, small size, and light quality. The data required to be processed are small in scale but rich in information. Combining big data analysis and AI can realize real-time automation applications [4]. Therefore, the MEMS sensors can be regarded as a more effective method of motion capture.

The existing motion recognition algorithms based on inertial sensors can be roughly divided into static recognition algorithms based on a single example and recognition algorithms based on time series according to whether time coherence information is considered. The actions in daily life are usually coherent and, thus, will be presented in the form of time series. The feature vectors with time series are used to describe human actions. The algorithm based on time series can recognize and analyze human daily actions through the transfer of action feature sequences. Due to the context-dependent pattern analysis, the recognition algorithm based on time series often has a good classification effect on the recognition of complex human actions, but the algorithm model is complex, and the model operation cost is high and it is difficult to realize real-time recognition when the data volume is large.

Although motion recognition algorithms based on inertial sensors have attracted the attention of researchers, most of the existing research focuses on distinguishing common simple motions, for example, researchers used the data of athletes collected by inertial sensors to identify the motions such as running, jumping, and walking [5]. A two-axis acceleration sensor is fixed at the joint of the subject to distinguish four types of standing, walking, lying, and sitting by the threshold method [6]. The threshold method is used to identify human falling motion by the triaxial acceleration sensor. But it is difficult to meet the task requirements of recognizing complex actions. In the research of actual sports, more attention is paid to the recognition of basic movements, which are mainly dependent on wrist sports. For example, Lin et al. [7] identified the motions with significant differences and characteristics in badminton, such as spike and catch. By using wireless wearable devices, researchers obtained the tennis action data of players completing the high balls, straight balls, and spikes [8], and then used multiple machine learning algorithms for recognition. However, there are insufficient researches on football and other sports, which are mainly composed of lower limb movements. Researchers used wearable devices to record the position of football players during the match and visualized their movement track by placing them on the thermal map [9], but did not analyze and process the individual movements of players. Rogalski et al. [10] proposed the relationship model between football players' training performance and injury risk by extracting training load data and physical attributes. Due to the complexity and variability of football actions and the high variability of individual execution, the trajectory of sports data is changeable, so it is difficult for the existing algorithm models to learn the data features directly. Moreover, most studies focus on distinguishing motions accurately and efficiently but rarely evaluate the completion quality of actions, which cannot meet the needs of football teaching and training.

**Figure 1:**
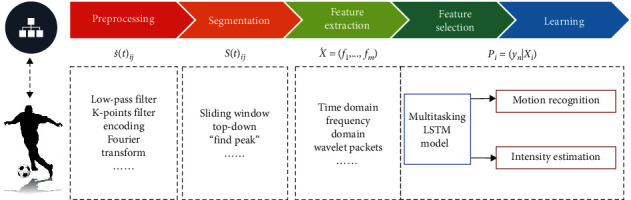
The framework of our proposed football motion recognition and intensity analysis system.

In this paper, as shown in Figure 1, a low-cost AI + IoT system framework is designed to recognize football motion and analyze motion intensity. To reduce the communication delay and the computational resource consumption caused by data operations, a multitask learning model is designed to achieve motion recognition and intensity estimation. The model can perform classification and regression tasks in parallel and output the results simultaneously. Compared with multiple models and methods, a single model has some major advantages: (1) simplicity: one can use this single model to obtain both classification and intensity results from the same time series data obtained from a sensor, and (2) cost-effective in resource constraint scenarios: for mobile and wearable platforms, a single model can reduce the execution time and power consumption of the task to recognize activity type and measure intensity. In addition, our approach leverages only simple sensor data such as acceleration and orientation to achieve this single model, instead of relying on video data. A feature extraction scheme is designed in the initial data processing, and feature data augmentation is performed to solve the small sample data problem. Experiments are conducted on the collected football motion datasets and the experimental results show that the designed multitask model can perform two tasks simultaneously and can achieve high computational efficiency. The multitasking single-layer long short-term memory (LSTM) network with 32 neural units can achieve the accuracy of 0.8372, F1 score of 0.8172, mean average precision (mAP) of 0.7627, and mean absolute error (MAE) of 0.6117, while the multitasking single-layer LSTM network with 64 neural units can achieve the accuracy of 0.8407, F1 score of 0.8132, mAP of 0.7728, and MAE of 0.5966.

## 3. Materials and Methods

### 3.1. System Design and Data Acquisition

**Figure 2:**
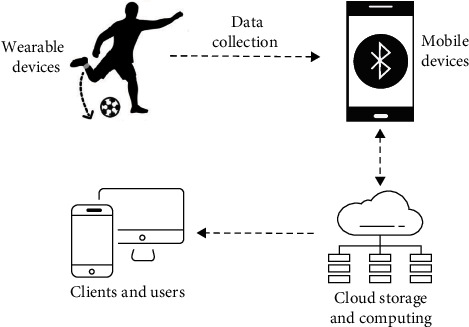
Illustration of the football motion recognition system.

In recent years, wearable sensors or devices have been widely used in motion recognition, but the existing application methods are difficult to be used in football motion recognition. Moreover, it is difficult for the existing limited number of coaches to carry out accurate quantitative data evaluation on the individual performance of each football player. First, a football motion recognition system based on IoT is introduced. The system can display sports types and sports performance to mobile devices in the form of professional visual data. This section describes the hardware design, interface software, and data acquisition process and details of each step.  Figure 2 shows the system framework, which consists of wireless wearable mobile devices, and a cloud-based data processing platform. Within the IoT system, low-power Bluetooth devices send motion data collected by wearable devices to mobile devices; when the mobile device receives the motion data, it transmits the original data to the remote service platform through cloud computing technology and completes all data collection and processing. Users can view the results of the analysis of the device user's performance through the platform.

#### 3.1.1. Hardware and Software System Design

**Figure 3:**
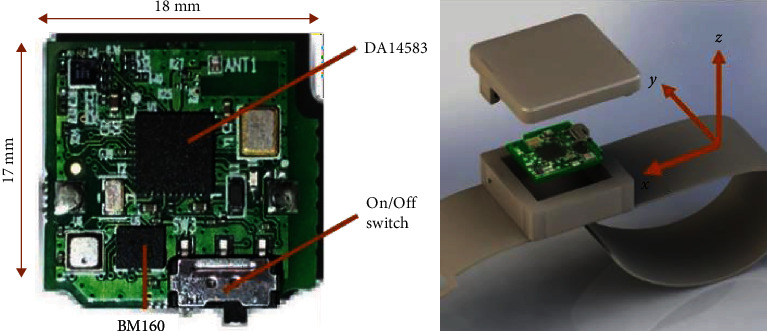
The structure of the wearable device.

The football motion recognition and evaluation system first collect data using a wireless wearable device.  Figure 3 shows the components of the device. The main components of the wireless wearable devices include a MEMS motion sensor chip with a three-axis gyroscope and accelerometer, a microprocessing unit with Bluetooth wireless communication function, a lithium battery, and a device switch. The three-axis gyroscope and accelerometer in MEMS are used to capture motion information and convert the signal into raw data. The device uses a BMI160 sensor from BOSCH. The microprocessing unit in the device, the DA14583, is a baseband radio processor from reading in the UK that fully integrates the low-power Bluetooth transceiver. Using the inertial measurement unit (IMU) chip, 3 days acceleration and angular velocity can be obtained from the BMI160 sensor with high integration and low-power consumption. The mobile application developed in this study is used to receive and visualize data transmitted by wearable devices in real time. The application includes three features: wireless connectivity, real-time capture and presentation of data, and cloud synchronization of data. All received data will be displayed synchronously and saved locally. The received data can also be saved to a remote server through the cloud method. Once the data collection process is completed, the users can also access the analysis results on the client side.

#### 3.1.2. Data Acquisition

This study conducted data collection experiments in the Youth Academy of the Chinese Football Association. A total of 11 male football players were recruited, including five professional players and six amateurs. Professional football players have represented their clubs in more than 10 national matches, while nonprofessional footballers are beginners in university. While conducting the basic football motions, a lightweight wireless wearable device was attached to the participant's right ankle to ensure the motion data was fully captured. Each participant made 20 inside foot passes and 20 arch shots on the football field. The subjects repeated these actions in the same position and required that both the pass and the shot be carried out with a certain speed and precision, otherwise it would be considered an invalid action, and no counting was done.

### 3.2. Feature Extraction and Data Enhancement

We first loaded the raw data from each subject. Then, we applied a three-point filter moving average to reduce the effect of noise and obtain a clearer signal. The statistical and morphology features were extracted and each dataset was merged into a large matrix. Segmentation was processed automatically by finding the peak of the signal. This window-based method can realize real-time data processing.

Several studies [11, 12] tended to use combined statistical features and frequency domain features to represent data features together to improve the quality of the feature vector. The key point of the quality of the feature vector is that it can represent the intrinsic features of each type of data.

In this section, a feature extraction method is designed to extract data streams from the original spectrum of 3D acceleration and gyroscope data as feature vectors for training the deep learning model. In addition to the few training data samples, the sensor is placed in the same part of the data collection process that will result in the displacement problem. The displacement problem is one of the reasons leading to the misclassification. Considering the problem of deviation of original data, it is a tedious process to correct by formulas and features. An effective solution to this problem is data enhancement. Data enhancement can generate the deformed dataset of the original data sample set without changing the semantics of the original data. The deformed part of the dataset can represent the deep feature information of the original data and thus can be used as a part of the training feature. Data enhancement is a popular method in image processing, in which image rotation and image scaling produce important semantics similar to the original image. However, for wearable sensor data, it is difficult to maintain the semantics of data labels using appropriate data enhancement methods, as changes in the raw data are a key factor in assessing the intensity of motion. Nevertheless, data enhancement has also been successfully applied to the study of wearable sensor data [13] and good performance in voice recognition [14]. Unlike the enhancement of image and speech recognition, the data enhancement of wearable sensor data needs further research. Therefore, in this study, a data enhancement scheme based on wireless wearable sensor data is designed. The data enhancement scheme is based on the local mean downsampling method. Finally, data cleaning is carried out to achieve a further local average of data and reduce overfitting effectively.

#### 3.2.1. Feature Extraction

**Figure 4:**
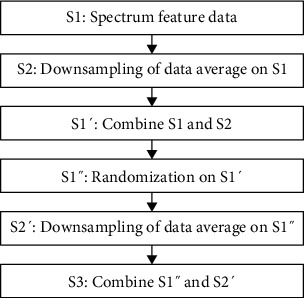
Feature extraction process.

Figure 4 shows the workflow of feature extraction. The short-time Fourier transform (STFT) extracted by each window can be generated by Equation (1).

(1) $$\begin{array}{c} \text{STFTk}\left[n\right]\left(m,\omega \right)=\sum_{-\infty }^{\infty }k\left[n\right]W\left[n-m\right]e^{-jn\omega }. \end{array}$$

In Equation (1), *k*[*n*] is the input signal and *W*[*n* − *m*] is the window function. To perform an STFT, the signal is first divided into overlapping windows of a fixed length. The choice of window length depends on the specific application and desired frequency resolution. A window function, such as the Hamming window, is then applied to each window to reduce spectral leakage caused by discontinuities at the edges of the window. Next, the Fourier transform is applied to each windowed segment of the signal, producing a complex-valued frequency-domain representation of the signal. Finally, the magnitude or power spectrum is calculated for each window and stacked together to form the spectrogram.

In each window, the power densities represented are combined and sorted in ascending order as the spectrum maps for each dimension are generated. Select a set of features that comprise a dataset consisting of a minimum power density and a set of power densities consisting of a maximum power density. Set the data of the minimum power density as *S* and the number of the maximum power density as *L*; the value of the acceleration sensor is *A*, and the value of gyroscope angular velocity is *G*. Combine S and L generated by gyroscope and acceleration as the input of model learning module. The data feature input is the spectrum information, as shown in Table 1.

**Figure 5:**
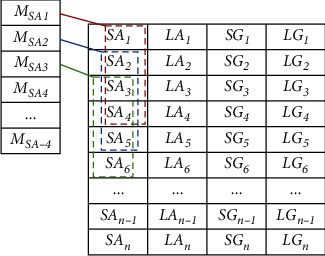
Continuous feature extraction of spectrum data based on sliding window.

The subsequent window of the data, as shown in Figure 5, is generated by overlapping 50% with the previous window in order to generate local average downsampling for continuous feature extraction. In this study, when *S* and *L* of spectral data of accelerometer and gyroscope are equal to 50, respectively, a dataset of 100 feature vectors is generated. In the same case, when *S* and *L* of accelerometer and gyroscope spectral data are equal to 100, respectively, a dataset of 200 feature vectors is generated. In subsequent experiments, the dataset of 100 feature vectors and 200 feature vectors is called the initial feature set.

The motivation to use minimum and maximum spectrum information here is that the original form of spectrum information can be used to generate features. For example, the convolution method was successfully used by Gu et al. [15] to generate features. In essence, convolution can extract significant feature representation data from the spectrum graph. Similarly, partial data can be selected as features using the same raw spectrum data without transformation, with boundary or limiting factors set. Compared with the process of selecting the minimum and maximum spectral information, the convolution process presents a larger delay, but in order to ensure the efficient real-time processing performance of the model, the factors causing the delay should be reduced as much as possible.

#### 3.2.2. Data Enhancement

If the initial feature dataset is regarded as a matrix of data points, each row represents a sample, including minimum acceleration data (*SAi*), maximum acceleration data (*LAi*), minimum gyroscope angular velocity data (*SGi*), and maximum gyroscope angular velocity data (*LGi*), where I is the sample number. Then, the data can form incremental data (*M*_SA1_, *M*_LA1_, *M*_SG1_, *M*_LG1_) by downsampling the average of each column (*M*_SAL_, *M*_LAL_, *M*_SGL_, *M*_LGL_). The new special solicitation is rearranged, and the obtained data is shown in Table 2.

**Figure 6:**
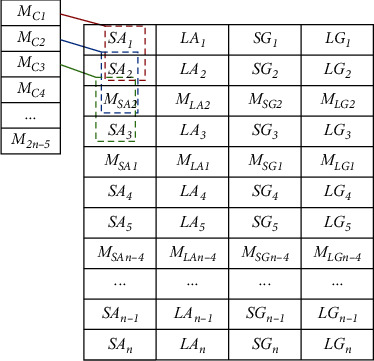
Incremental data with feature vectors (MC11, MC12, MD11, MD12) generated by downsampling.

Then, through continuous averaging of two column items of all columns, downsampling is conducted again to generate incremental data with feature vectors (MC11, MC12, MD11, MD12), as shown in Figure 6.

It is attached to the random dataset represented in Table 3. In the process of processing small-batch data, random sorting of data can reduce data differences, keep the model universal, and reduce overfitting [16]. In the experiment, an interline randomization method was used for each category.

### 3.3. Motion Recognition and Intensity Estimation Based on LSTM

**Figure 7:**
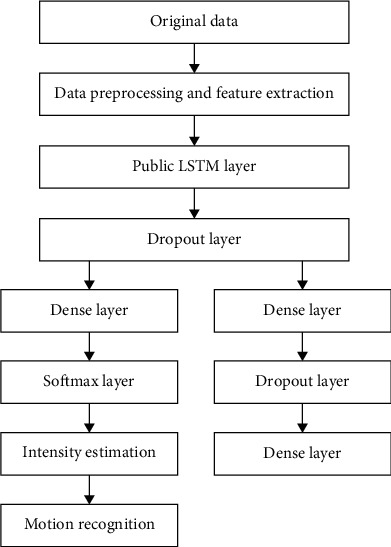
Multitask model based on LSTM.

In this section, we propose a multitask learning model based on LSTM. The data collected from the wearable sensor itself capture the type of motion and intensity of motion, so both tasks can be accomplished simultaneously through data-level fusion. The model uses raw wearable sensor data as input to classify activity types and assess exercise intensity.  Figure 7 shows the overall architecture of the multitasking model.

The subtasks can be divided into classification tasks and regression tasks according to the output results of the tasks performed by two nonoverlapping learning channels.

#### 3.3.1. Model Design

The first LSTM layer in the model extracts a fresh set of motion data features, which are explored by the next two subtask branches of the model to assist each subtask model learning, and the original data features are fed into each branch to regulate learning. In the subtask model of motion type classification, a fully connected output layer with Softmax uses these new features to produce the class probabilities; in the subtask model of intensity estimation, data features are input to the full connection layer of another partial neural network, and the network has a certain neural unit discard rate to prevent the overfitting problem of prediction, so as to estimate the target intensity value of prediction category.

The LSTM layer is selected as the feature extractor in the model. The LSTM layer encodes the sequential motion data through the temporal data features. *T* = [*r*(1, *m*), *r*(2, *m*),…, *r*(*n*, *m*)] as an input matrix of size *n* × *m*, where *m* is the dimension size of each axis sampled by a wearable sensor, and *n* is the number of data points during a time interval. A multiple dimensional reproduction scheme on the original dataset is presented, and the number of hidden units is given. The adopted LSTM takes the raw sequential data as input and conduct the feature extraction on hidden layers. The specific layers of the adopted LSTM are stackable, so that the output layer of the LSTM layer can be used as the input layer of the next LSTM layer, which in turn is superimposed to form a deeper model. A coded representation vector *V* = [*v*(1, *m*), *v*(2, *m*),…, *v*(*n*, *m*)] is produced by the LSTM layer. Its size equals to the size of the original input, and the output results are sent to the other LSTM layer for extracting more hidden information.

As for the classification task, the full connection layer is used to map the multidimensional representation vector to *X* ∈ *C*, where *C* is the number of motion classes. In the intensity estimation task, another full connection layer is added for further extracting the data features, so as to obtain a higher evaluation accuracy in the intensity estimation. The resulted model is an integrated multitasking network with features from a common LSTM layer being channeled into subtask models with full connectivity layers.

The loss function of motion classification is computed by the cross-entropy loss function of classification given in the Keras library, and the specific Equation (2) is shown as follows:

(2) $$\begin{array}{c} Lc=-\frac{1}{N}\sum_{i=1}^{N}\sum_{c=1}^{C}1_{y_{i}\in C_{c}}\log\left(p_{model}\left[y_{i}\in C_{c}\right]\right), \end{array}$$

where *Lc* denotes the loss value of the classification model, *N* denotes the size of batch, and *C* is the number of motions classes. In Equation (2), 1 denotes the value of indicator function, and log(*p*[*y*_*i*_]) denotes the logarithmic probability that a given input *Y*_*i*_ belongs to class *C* when mapping with model *p*. The loss function of mean square error (MSE) is a more appropriate for the regression model for intensity estimation task, because the output numerical result should be a nonnegative integer value, which is given in Equation (3):

(3) $$\begin{array}{c} L_{i}=-\frac{1}{N}\sum_{i=1}^{N}{\left(y_{i}-\bar{y_{i}}\right)}^{2}, \end{array}$$

where *L*_*i*_ denotes the estimated loss, and *y*_*i*_ denotes the accelerometer value for each of the three axes. For the multitask model, the final loss function was obtained by weighting the loss of motion classification and exercise intensity estimation. The final loss function is given in Equation (4) that is shown as follows:

(4) $$\begin{array}{c} L=\alpha_{c}\;L_{c}+\alpha_{i}\;L_{i}, \end{array}$$

where *L* is the value of the total loss function, *α*_*c*_ denotes the weighted coefficient of the classification loss *Lc*, and *α*_*i*_ denotes the weighted coefficient of the strength estimation loss *L*_*i*_. The multitask LSTM model can be used to accomplish two different tasks simultaneously.

#### 3.3.2. Model Optimization

As the size of the training dataset is small, overfitting is the major problem in model training. Regularization methods on L1 and L2 are used to cut down the model overfitting effects. Some neural units were randomly discarded after each layer of the model, and the discard rate of neural units was 0.5. All rectified linear units (ReLUs) are used as the activation function for all hidden layers. In the output layer, the Softmax function is used to obtain class probabilities in classification problems. For the separation of testing and trainning datasets, cross-validation techniques are used, and the final results of the model are obtained by averaging the results of all subjects.

## 4. Results and Discussion

This section introduces the experimental results of the training and testing phases of the multitasking LSTM model. For the training stage, model parameters are debugged, such as the number of input channels and LSTM layers. First, the single-task independent model of motion classification and intensity estimation is trained through the training dataset. During this stage, the optimal model parameters and hyperparameters are obtained. Then, the multitasking model is trained, and the training results of the multitasking model are compared with the output results of multiple single-task models. Then, the optimal structure of the multitask learning model is obtained by testing a different number of LSTM layers. First, the data of all participants were combined. Then, samples were randomly extracted from the combined dataset and the test set are partitioned. The model was cross-verified repetitively.

### 4.1. Experimental Design

Two hundred sixty-four groups of passing and 250 groups of shooting data were obtained through a data acquisition experiment. The dataset was randomly divided into the training set and test set, 80% of which was used for training and the rest for testing data. To verify the training model, the batch size was selected as 300 and the model was iterated 14,000 times. In addition, the input data is normalized. The Keras library provides several optimization techniques, including stochastic gradient descent (SGD), RMSProp, and Adam optimizer, which was selected for model optimization in experiments. The weighted coefficient of the loss function *L*_*c*_ of the classification task submodel was set as *α*_*c*_ = 1. The weighted coefficient *α*_*i*_ of the loss function *L*_*i*_ of the motion intensity estimation subtask is 0.001, because at the beginning of multitask network training, the loss function of the motion intensity estimation subtask model is usually about 6,000, while the loss function of the motion classification subtask model is about 3.5. When the motion completion rate is directly used as the output of the model, the loss *L*_*i*_ of the motion intensity estimation subtask model is weighted to 0.1.

F1 scores and mAP were used to assess the quality of the motion classification subtask model, and mean absolute percentage error (MAPE) was used to estimate the intensity of the motion intensity estimation submodel for each activity type. The slight overfitting of the model is due to the nature of the method and the limited amount of data. According to Barut et al. [17], after parameter grid search, the final hyperparameters of the optimal model setting are given as follows: learning rate is set as 0.0002, weight attenuation is 0.00001, neural unit discard rate is set as 0.5, the batch size is 300, the iteration number is set as 50,000, and regularization constant is set as 0.0002. In the optimal structure, the hidden units for a single LSTM layer is 32 single-layer models, since a small number of hidden units lack of learning ability for feature information, while a large number of hidden units can only improve the metric of F1 score, but greatly increase the computational cost.

### 4.2. Experimental Results and Analysis

The results of action recognition subtasks in the multitask deep learning model are shown in the two right-most columns, as shown in Table 4. The worst performing model in the classification task was the one using the two-layer LSTM with 16 neural units as the complexity of the model was not enough to capture different features, while the two-layer LSTM with 64 neural units was too complex. The best performing model was the single-layer LSTM with 64 neural units as it achieved the highest accuracy in both the training and test sets.

In the experiment, models with different numbers of hidden units and layers were compared, and the output effect of the model with an additional fully connected layer was tested. The results are shown in Table 5. It can be found that adding a full connection layer can reduce the MAE in the mean of cross-validation results. In addition, it is not difficult to see that the use of more hidden units helps the model better map the input wearable data to count estimates, thus improving the accuracy of motion intensity estimation.

The optimal model of motion intensity estimation in the single-task model is realized by a two-layer LSTM network with 64 neural units and a full connection layer with 32 neural units added. The experimental results are given in the last line of the table. The two-layer LSTM layer can extract more information-containing features from the motion intensity estimation subtask model, but the single-layer LSTM network with 64 neural units and a full connection layer can achieve the performance with lower complexity, and the single-layer model can obtain the highest average F1 score in the motion classification model. The average F1 score of the motion classification task submodel is 0.8106.

The performance of the comprehensive multitask model is compared with the results of the single-task model. To understand the performance of the multitasking model, F1 scores and mAP indicators of the multitasking model were compared with those of the single-tasking model, and the results are shown in Table 6.

For the motion classification task, the single-task single-layer LSTM network with 64 neural units without a full connection layer got the highest score on all indicators. In the motion intensity estimation, the single-task and single-layer LSTM model with 64 full connection layer neural network units obtained the lowest error value of MAE. However, the model also achieved the lowest error in measuring MAPE when it adopted the model scale with 32 neural units. Compared with the model with 64 neural network units, its complexity was lower and the degree of overfitting was lower, so the outliers with the highest MAPE error were eliminated. At the same time, the model has the performance of motion classification with high accuracy. This suggests that using one multitask model can improve the performance of the motion intensity estimation task better than using multiple single-task models, but this requires a slight cost in the accuracy of the motion classification task. By means of the moving average filter and so on, the data noise can be reduced and the data flow can be smoothed. The model performs well on real datasets, but the data scale limits the model's performance to a certain extent. Therefore, feature enhancement and model training parameter adjustment is adopted in this paper to avoid overfitting the model, and data are continuously collected in subsequent applications. Expanding the data scale can help improve the model's identification accuracy. Moreover, we have compared our algorithm with other models, as shown in Table 7.

In addition to comparing the performance of multiple single-task and multitask models, different datasets were used to evaluate the models, including UCI-HAR [18], BaSA [19], and Stanford-ECM [20], to better verify whether the multitask model is based on sensor data can improve the performance of motion classification tasks.  Table 8 summarizes the results obtained by the model.

First, the total acceleration (A) rate is extracted from the UCI-HAR dataset as a data feature. The classification accuracy obtained by the single-task model using this dataset indicates that the model performs well, but not as well as the results of the model proposed in the latest study. Therefore, the gyroscope (G) data was added to increase the characteristic data information and retrain the model. However, the model obtained no better results after the gyroscope data was added than the model using only the acceleration data. Compared to the single-task model, the performance of the multitask model is reduced because the model combines two independent tasks in one task and uses acceleration counting and gyroscope data for direct estimation at the input end. However, the recognition rate of the action classification subtask model in the multitask model is obviously improved and exceeds that of the single-task model. However, in our dataset, the precision of motion classification of all models can be improved by using gyroscope data and acceleration data as input.

The multitask model is trained on the BaSA dataset to understand the performance of the multitask model when dealing with multisensor data problems. The data was lowered to 50 Hz to train the model. The results show that the model can achieve accurate results in the classification of motion, but the estimation of motion intensity is not so accurate, because the multitask model tends to improve the classification accuracy at the expense of the estimation of motion intensity.

Finally, the multitask model is evaluated for a subset of the Stanford-ECM dataset. Merge data features in the dataset named TalkingSitting and SittingTasks into Sitting, and data features TalkingStanding and StandingInLine into Standing. Six categories were extracted from the original dataset (Sitting, Standing, Walking, Running, DescendingStairs, AscendingStairs). The submodel of the intensity estimation task got an excellent MAE of 0.229. However, the submodel of the motion classification task does not perform well in mAP. Because of the class imbalance in the Stanford-ECM6 dataset, the model has a large deviation with a small variance.

## 5. Conclusions

This paper constructs an algorithm for the football movement recognition system based on the IoT devices. The system can identify the user's football movement and evaluate the quality of the movement. In the core algorithm model of the research system, the designed multitask LSTM motion classification and motion intensity estimation model are introduced, which mainly includes two parts: original data processing and multitask learning model design. According to the original IMU sensor data, a feature extraction method based on the spectrum is designed. By sorting and extracting a set of defined minimum and maximum values, the length of the data stream is reduced while the effective information in the data is kept as far as possible. On this basis, feature data is enhanced. This data processing method can create new data features of training samples without changing the semantics of the original data. The model is used for motion classification and motion intensity estimation tasks based on data obtained from a single wearable device. The model has two learning channels, and the two tasks are carried out in parallel in a learning model at the same time, and the results of the classification task and regression task are output simultaneously. In order to verify the feasibility of the model, the multitask model is compared with multiple single-task models in the verification experiment. Then, experiments are carried out on three public datasets, and the performance and learning mode of the model are analyzed. However, for commercial applications, real-time analysis is very important. Therefore, we will improve our sensing and analyses system by collecting wireless data in real-time in the future.

## Figures and Tables

**Table 1:** Spectrum data acquisition based on IMU sensor data.

*SA*_*1*_	*LA*_*1*_	*SG*_*1*_	*LG*_*1*_
*SA*_*2*_	*LA*_*2*_	*SG*_*2*_	*LG*_*2*_
*SA*_*3*_	*LA*_*3*_	*SG*_*3*_	*LG*_*3*_
*SA*_*4*_	*LA*_*4*_	*SG*_*4*_	*LG*_*4*_
…	…	…	…
*SA*_*n−1*_	*LA*_*n−1*_	*SG*_*n–1*_	*LG*_*n−1*_
*SA*_*n*_	*LA*_*n*_	*SG*_*n*_	*LG*_*n*_
Maximum and minimum spectrum data of accelerometer		Maximum and minimum spectrum data of gyroscope	

IMU, inertial measurement unit.

**Table 2:** The obtained new special solicitation by downsampling.

*SA*_*1*_	*LA*_*1*_	*SG*_*1*_	*LG*_*1*_
*SA*_*2*_	*LA*_*2*_	*SG*_*2*_	*LG*_*2*_
*M*_*SA2*_	*M*_*LA2*_	*M*_*SG2*_	*M*_*LG2*_
*SA*_*3*_	*LA*_*3*_	*SG*_*3*_	*LG*_*3*_
*M*_*SA1*_	*M*_*LA1*_	*M*_*SG1*_	*M*_*LG1*_
*SA*_*4*_	*LA*_*4*_	*SG*_*4*_	*LG*_*4*_
*SA*_*5*_	*LA*_*5*_	*SG*_*5*_	*LG*_*5*_
*M*_*SAn−4*_	*M*_*LAn−4*_	*M*_*SGn−4*_	*M*_*LGn−4*_
…	…	…	…
*SA*_*n−1*_	*LA*_*n−1*_	*SG*_*n−1*_	*LG*_*n−1*_
*SA*_*n*_	*LA*_*n*_	*SG*_*n*_	*LG*_*n*_

**Table 3:** Datasets randomization.

*SA*_*1*_	*LA*_*1*_	*SG*_*1*_	*LG*_*1*_
*SA*_*2*_	*LA*_*2*_	*SG*_*2*_	*LG*_*2*_
*SA*_*3*_	*LA*_*3*_	*SG*_*3*_	*LG*_*3*_
…	…	…	…
*M*_*SAn−4*_	*M*_*LAn−4*_	*M*_*SGn−4*_	*M*_*LGn−4*_
…	…	…	…
*SA*_*n−1*_	*LA*_*6*_	*SG*_*6*_	*LG*_*6*_
*SA*_*n*_	*LA*_*7*_	*SG*_*7*_	*LG*_*7*_
*M*_*C11*_	*M*_*C12*_	*M*_*D11*_	*M*_*D12*_
*M*_*C21*_	*M*_*C22*_	*M*_*D21*_	*M*_*D22*_
…	…	…	…
*M*_*CK1*_	*M*_*CK2*_	*M*_*DK1*_	*M*_*DK2*_

**Table 4:** Motion recognition comparison between L1 and L2.

Model	F1 score			mAP	
	Subject 1	Subject 2	All sample data	Training dataset	Testing dataset
L1 : 16	0.7242	0.8538	0.7462	0.9095	0.9225
L1 : 32	0.9260	0.9058	0.8261	0.9643	0.9571
L1 : 64	0.7866	0.9398	0.8343	0.9933	0.9776
L2 : 16	0.7438	0.8066	0.6799	0.8303	0.8633
L2 : 32	0.7431	0.8588	0.7721	0.9138	0.9291
L2 : 64	0.7828	0.9667	0.8046	0.8932	0.8843

**Table 5:** Intensity estimation comparison between L1 and L2.

Model	Fully connected layer	MAE		
		Subject 1	Subject 2	All sample data
L1 : 32	0	2.371	1.429	1.624
L1 : 32	1	1.163	1.576	0.931
L1 : 64	0	1.052	1.093	0.857
L1 : 64	1	0.711	0.582	0.569
L2 : 32	0	1.156	0.519	0.627
L2 : 32	1	1.640	0.687	0.632
L2 : 64	0	0.711	0.658	0.586
L2 : 64	1	0.573	0.631	0.546

**Table 6:** Results comparison among several models.

Model	Accuracy	F1 score	mAP	MAE	MAPE (%)
Single-task single-layer LSTM (32)	0.8419	0.8261	0.7681	0.9313	37.36
Single-task single-layer LSTM (64)	0.8566	0.8343	0.7913	0.5690	18.51
Multitask single-layer LSTM (32)	0.8372	0.8172	0.7627	0.6117	17.19
Multitask single-layer LSTM (64)	0.8407	0.8132	0.7728	0.5966	22.34

LSTM, long short-term memory.

**Table 7:** Results comparison among several models.

Model	Accuracy	F1 score
SVM	0.8419	0.8261
KNN	0.8566	0.8343
CNN	0.8372	0.8172
This study	0.9407	0.8432

**Table 8:** Experimental results of multitask models on UCI-HAR public datasets.

Model	Sample data type	Clsf. (mAP)	Reg. (MAE)
UCI: Single task	A	0.889	–
UCI: Single task	A, G	0.880	–
UCI: LSTM	A, G	0.908	–
UCI: LSTM-multitask	A, G	0.935	–
BaSA: Single task	A, G	0.732	1720
BaSA: LSTM	A, G	0.796	3,230
BaSA: LSTM-multitask	A, G	0.803	5,000
ECM: Single task	A	0.346	0.229
ECM: LSTM	A	0.523	0.768
ECM: LSTM-multitask	A	0.781	0.696

LSTM, long short-term memory.

## Data Availability

Data deposited in a repository of https://github.com/AlgLab/SCMU-football-motion-dataset.
